# Recursive partitioning analysis of patients with oligometastatic non-small cell lung cancer: a retrospective study

**DOI:** 10.1186/s12885-019-6216-x

**Published:** 2019-11-06

**Authors:** Jia-Tao Zhang, Si-Yang Liu, Hong-Hong Yan, Yi-Long Wu, Qiang Nie, Wen-Zhao Zhong

**Affiliations:** Guangdong Lung Cancer Institute, Guangdong Provincial People’s Hospital, Guangdong Key Laboratory of Lung Cancer Translational Medicine, South China University of Technology & Guangdong Academy of Medical Sciences, Guangzhou, 510080 China

**Keywords:** Oligometastasis, Local consolidative therapy, Recursive partitioning analysis, Prognosis risk stratification

## Abstract

**Background:**

Local consolidative treatment (LCT) is important for oligometastasis, defined as the restricted metastatic capacity of a tumor. This study aimed to determine the effects and prognostic heterogeneity of LCT in oligometastatic non-small cell lung cancer.

**Methods:**

This retrospective study identified 436 eligible patients treated for oligometastatic disease at the Guangdong Provincial People’s Hospital during 2009–2016. A Cox regression analysis was used to identify potential predictors of overall survival (OS). After splitting cases randomly into training and testing sets, risk stratification was performed using recursive partitioning analysis with a training dataset. The findings were confirmed using a validation dataset. The effects of LCT in different risk groups were evaluated using the Kaplan-Meier method.

**Results:**

The T stage (*p* = 0.001), N stage (*p* = 0.008), number of metastatic sites (*p* = 0.031), and EGFR status (*p* = 0.043) were identified as significant predictors of OS. A recursive partitioning analysis was used to establish a prognostic risk model with the following four risk groups: Group I included never smokers with N0 disease (3-year OS: 55.6%, median survival time [MST]: 42.8 months), Group II included never smokers with N+ disease (3-year OS: 32.8%, MST: 26.5 months), Group III included smokers with T0–2 disease (3-year OS: 23.3%, MST: 19.4 months), and Group IV included smokers with T3/4 disease (3-year OS: 12.5%, MST: 11.1 months). Significant differences in OS according to LCT status were observed in all risk groups except Group IV (*p* = 0.45).

**Conclusions:**

Smokers with T3/4 oligometastatic non-small cell lung cancer may not benefit from LCT.

## Background

A comprehensive review of metastasis theory before 1995 reveals two main mechanisms, Halsted’s theory and the systemic hypothesis, which were addressed using local and systemic treatment, respectively [1–4]. In 1995, Hellman and Weichselbaum proposed a clinically significant state of metastasis, “oligometastasis,” which refers to a restricted tumor metastatic capacity [5]. This clinical entity was initially considered an intermediate state of metastatic evolution, and local treatments were considered potentially curative in this setting [6–8]. However, the precise definition of oligometastasis remains highly uncertain, which ultimately affects our understanding of the mechanisms underlying cancer metastasis. However, a diagnosis of oligometastasis mainly relies on the observation of a change after systemic therapy, and the number of metastatic sites (either 1–3 or 1–5) is considered the main determinant.

Lung cancer is the leading cause of cancer-related death worldwide, and more than 700,000 new cases are diagnosed each year in China [9, 10]. Over the past decade, studies concerning oligometastasis have identified various factors that can predict a favorable prognosis and support an indication for local therapy [7, 11–13]. The first randomized phase 2 study on this topic, which investigated local consolidative therapy (LCT) for oligometastatic non-small cell lung cancer (NSCLC), was reported in 2016 and revealed median progression-free survival (PFS) durations of 11.9 months in the LCT group and 3.9 months in the maintenance treatment group [14]. In other words, LCT appears useful for improving the prognosis of patients with oligometastasis, although several problems (e.g., predictive factors and treatment timing) must be resolved before this approach can be implemented in clinical practice.

Previous studies have indicated that the stable progression-free period after first-line systemic therapy may be the optimal window for LCT. Therefore, the present study aimed to evaluate the prognostic heterogeneity and factors related to LCT in patients with oligometastatic NSCLC.

## Methods

This retrospective study evaluated data from patients with oligometastatic NSCLC who were treated at Guangdong Provincial People’s Hospital. The retrospective study protocol was approved by the ethics committee of Guangdong Provincial People’s Hospital.

### Patient selection and LCT definition

In this report, we define oligometastasis as stage IV disease with ≤3 metastases, not including the primary tumor, based on the 7th edition of the TNM system. And the detailed inclusion criteria as follow: (1) pathologically confirmed NSCLC, (2) stage IV disease based on the 7th TNM staging system, (3) ≤3 synchronous or metachronous metastases (not including the primary tumor), (4) an Eastern Cooperative Oncology Group performance status of ≤2, and (5) a history of first-line systemic therapy (≥2 cycles of platinum-based chemotherapy or ≥ 1 month of EGFR/ALK targeted therapy). The number of metastatic sites was assessed using systemic imaging, namely computed tomography (CT), electrical capacitance tomography, or positron emission tomography (PET)-CT of the chest and abdomen and CT, magnetic resonance imaging, or PET-CT of the brain. Patients with pleural, pericardial, and meningeal metastases were excluded because the metastatic lesions could not be counted separately.

The decision to perform LCT was made by a panel of clinicians, including a thoracic surgeon, radiologist, and medical oncologist. LCT was defined as treatment with the intent to ablate all residual disease (primary tumor, lymph nodes, and metastatic sites) comprising surgery, radiotherapy, or both. The treating radiotherapists made decisions about any dose-fractionation regimen with curative intent when possible, although palliative intent was considered acceptable.

### Study design

A random number was assigned to each case according to the principle of simple randomization. Based on the numerical order of the random numbers, patients in the first half were grouped as the training set; the remaining patients were grouped as the validation set. A recursive partitioning analysis (RPA) based on the patients’ demographic and clinical characteristics was then performed with the intent to create a decision tree model that would correctly stratify risk in the target population [15]. The model was subsequently evaluated using the validation dataset. The effects of LCT were also investigated in various risk groups.

### Statistical methods

Associations between clinical characteristics were evaluated using the chi-square test. The Kaplan–Meier method and log-rank test were used to evaluate differences in overall survival (OS) and PFS. For the RPA, the recursive decision tree was created using free software (R version 3.3.2; rpart package version 4.1–11, http://www.r-project.org/) and was pruned by complexity parameter.

## Results

**Fig. 1:**
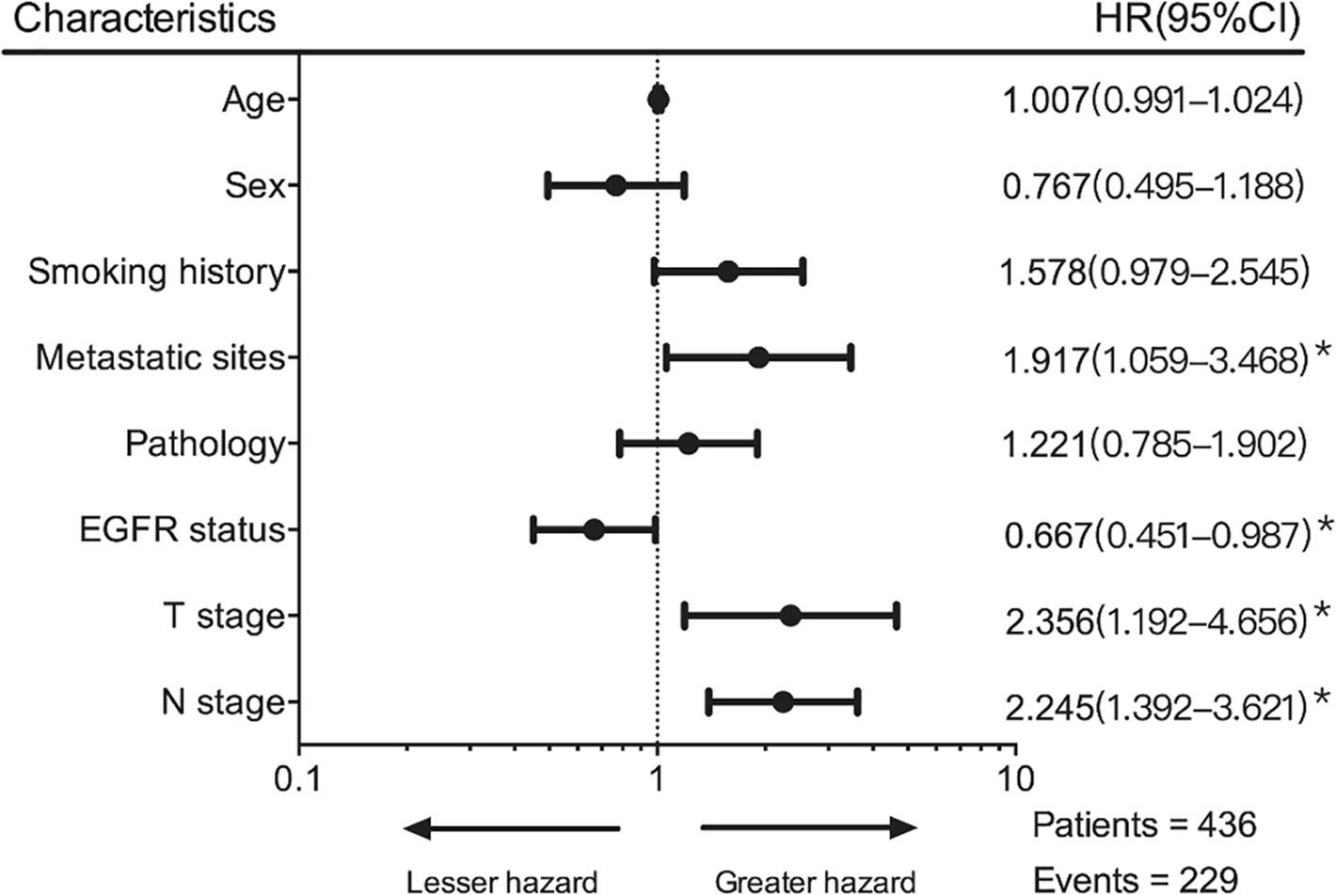
Cox regression analyses of risk factors in patients with oligometastatic non-small cell lung cancer. Dots indicate the unadjusted hazard ratios, horizontal lines indicate the 95%s confidence intervals, and asterisks indicate significant variables (*p*-value < 0.05)

This study included 436 patients with oligometastatic NSCLC who were treated during 2009–2016. The baseline characteristics from the training and validation datasets are shown in Table 1. The two datasets only differed significantly in terms of sex. Figure 1 presents the results from the Cox regression analysis of all patients, which revealed associations of an inferior outcome with the number of metastatic sites (hazard ratio [HR]: 1.91, *p* = 0.031), T stage (HR: 2.36, *p* < 0.001), and N stage (HR: 2.25, *p* < 0.001). Furthermore, an improved outcome was associated with EGFR mutation (HR: 0.67, *p* = 0.043).

**Table 1:** Baseline characteristics from the training and validation datasets

	Training	Validation	*P*-value
Age, years (range)	60.4 (36–88)	60.4 (31–84)	0.331
Sex, n (%)			0.045
Male	164 (75.2%)	144 (66.1%)	
Female	54 (24.8%)	74 (33.9%)	
Smoking history, n (%)			0.101
Never	112 (51.4%)	130 (59.6%)	
Former/current	106 (48.6%)	88 (40.4%)	
Metastatic sites, n (%)			0.233
1–2	208 (95.4%)	201 (92.2%)	
3	10 (4.6%)	17 (7.8%	
Classification, n (%)			0.690
Synchronous	137 (62.8%)	142 (65.1%)	
Metachronous	81 (37.2%)	76 (34.9%)	
Pathology, n (%)			0.616
Adenocarcinoma	164 (75.2%)	171 (78.4%)	
Non-adenocarcinoma	47 (21.6%)	45 (20.6%)	
Unknown	7 (3.2%)	2 (1.0%)	
EGFR status, n (%)			0.911
Negative	110 (50.5%)	107 (49.1%)	
Positive	77 (35.3%)	82 (37.6%)	
Unknown	31 (14.2%)	29 (13.3%)	
T stage, n (%)			0.851
T0–2	149 (68.3%)	155 (71.1%)	
T3–4	67 (30.7%)	61 (27.9%)	
N stage, n (%)			0.282
N0	49 (22.5%)	63 (28.9%)	
N+	169 (77.5%)	155 (71.1%)	
LCT			0.968
Surgery	17 (7.8%)	15 (6.9%)	
Radiotherapy	79 (36.2%)	80 (36.7%)	
Both	4 (1.8%)	3 (1.4%)	
Neither	118 (54.2%)	120 (55.0%)	
Total	218	218	

*EGFR* epidermal growth factor receptor; *LCT* local consolidative therapy

**Fig. 2:**
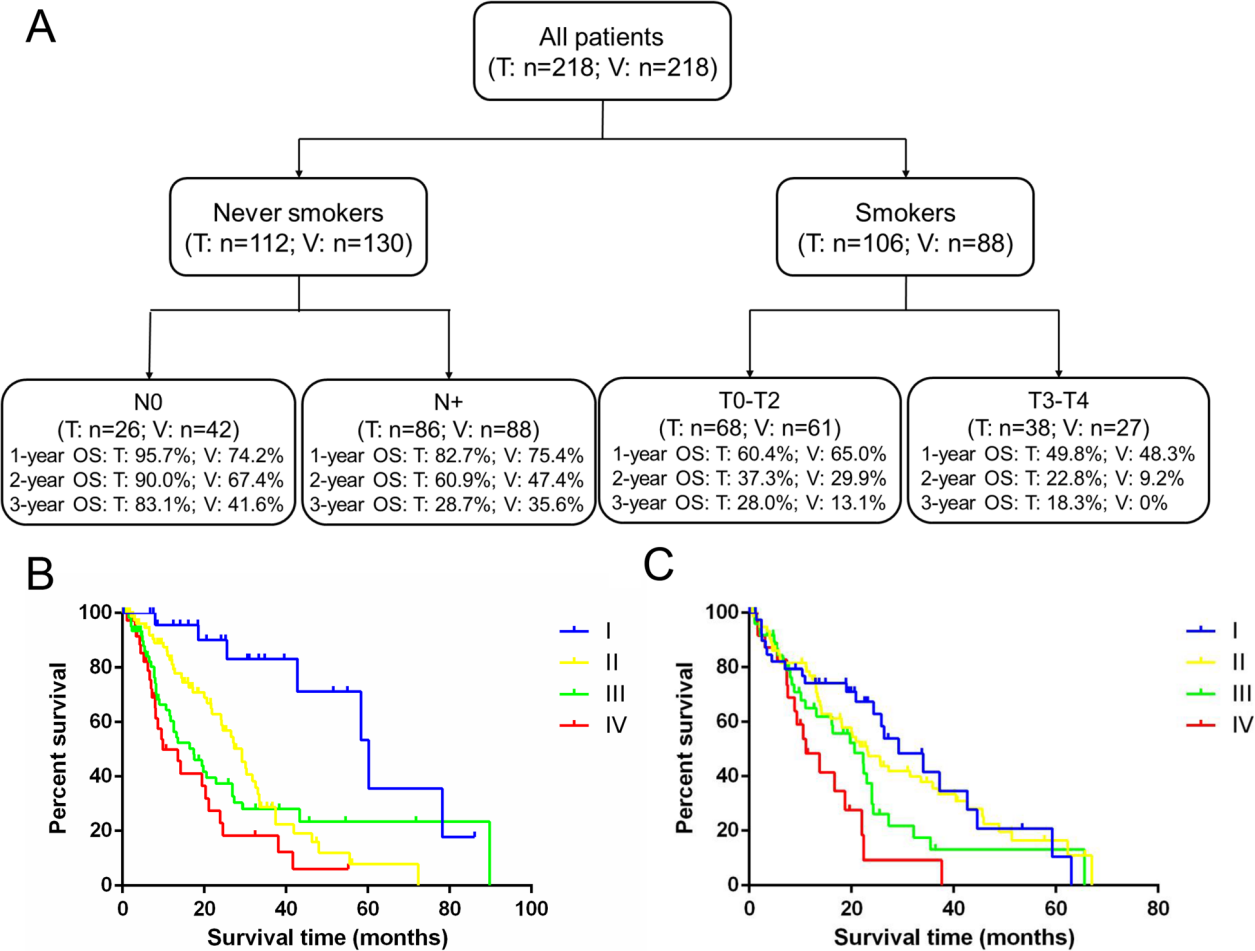
Flowchart of the recursive partitioning analysis. **a** The recursive partitioning analysis of patients with oligometastatic non-small cell lung cancer (T: training set, V: validation set). **b** The survival curves for the training dataset. **c** The survival curves for the validation dataset

The results of the RPA model for OS are shown in Fig. 2a. Based on the training dataset, the patients were divided into four risk groups: Group I included never smokers with N0 disease (3-year OS: 83.1%), Group II included never smokers with N+ disease (3-year OS: 28.7%), Group III included smokers with T0–2 disease (3-year OS: 28.0%), and Group IV included smokers with T3/4 disease (3-year OS: 18.3%). The various risk groups had significantly different 3-year OS rates (*p* < 0.001). The survival curves for the RPA model in the training and validation datasets are shown in Fig. 2b and c, respectively.

**Fig. 3:**
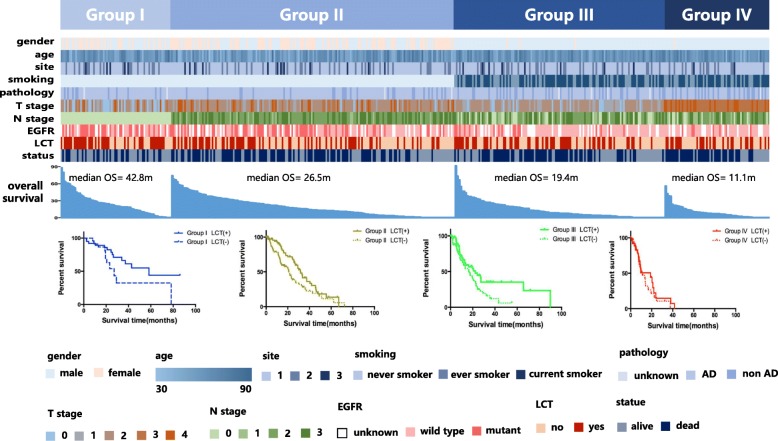
Distribution of patients’ characteristics according to the recursive partitioning analysis and the effects of local consolidative treatment in different risk groups

Figure 3 presents the clinical characteristics of all patients according to the RPA model and the survival curves of each risk group according to LCT status. Significant differences in the survival curves according to LCT status were observed for all risk groups except Group IV (*p* = 0.45), indicating that LCT provided limited survival benefits in this group of patients with oligometastatic NSCLC.

## Discussion

A considerable amount of literature has been dedicated to oligometastasis, and the importance of LCT in this context has gradually been accepted [16–21]. However, various problems must be addressed before specific patients with oligometastatic NSCLC who are expected to benefit from LCT can be identified. Therefore, the present study used RPA to examine the effects and prognostic heterogeneity of LCT for oligometastatic NSCLC. Our results indicate that patients who smoked and had T3/4 oligometastatic NSCLC would not be expected to benefit from LCT. However, the EGFR status was the only molecular characteristic considered in this study. As 60 patients (13.8%) did not undergo the related test, we cannot definitively comment on the contributions of molecular features in this group of patients. Moreover, in clinical practice, patients with a history of smoking and a large lung tumor typically have squamous cell carcinoma or a tumor without driver gene mutations, which suggests the presence of an unknown molecular mechanism that should be examined in future studies.

As shown in Table 1, sex characteristics were slightly imbalanced between the two datasets (*p* = 0.045). However, after careful consideration, we chose to accept this slight imbalance for the following reasons: 1) the relevant factors that could be affected by gender, such as pathology, EGFR status, and smoking history, were completely balanced between the two groups; 2) gender was not a statistically significant factor in the Cox regression model; 3) we believed that the slight gender difference would be acceptable and the subsequent RPA model could be verified in the two datasets; and 4) we used simple randomization in this study, which is likely to lead to this type of issue.

At the 60th ASTRO annual meeting, Professor Gomez presented the final results of a phase 2 trial that compared LCT to standard maintenance treatment or observation alone for patients with oligometastatic NSCLC. During a median follow-up period of 38.8 months (range: 28.3–61.4 months), the median OS was significantly longer in the LCT group than in the control group (41.2 months [95% CI: 18.9 months–not reached] vs. 17.0 months [10.1–39.8 months]; *p* = 0.017) [22]. Interestingly, in our study, patients in Group IV had a median OS of 11.1 months, similar to that in the control group. Conversely, Group I had a median OS (42.8 months) similar to that in the LCT group. These preliminary results support our main conclusion that patients in our Group IV (smokers with T3/4 disease) may not benefit from LCT.

The present study excluded patients with pleural, pericardial, and meningeal metastasis because these metastases could not be counted separately. Moreover, evidence suggests that pleural or meningeal metastases are disease entities with unique biological behaviors. For example, Zhong et al. reported that patients with intrathoracic disseminated pT4-M1a pleural metastases had a favorable prognosis [23]. In addition, several studies have indicated that limited surgery might be a good choice for the pleural dissemination of lung cancer [20, 24, 25]. However, meningeal metastasis, and especially leptomeningeal metastasis, is a different disease entity with a poor prognosis. Few studies have investigated the comprehensive profile of meningeal metastasis, for which no treatment strategies have been established. Li et al. recently reported that leptomeningeal metastasis was much more common in patients with EGFR-mutant NSCLC, who responded relatively well to EGFR-TKIs [26]. Therefore, we believe that our exclusion of patients with pleural, pericardial, and meningeal metastasis was reasonable.

The present study had two major limitations. First, a retrospective design is associated with a risk of selection bias. The selection of LCT for oligometastatic patients may have been an additional source of bias. Second, censoring the data may have confounded the results of our analyses. Moreover, we intended to base the RPA tree on the most obvious prognostic differences, which could have introduced some degree of ambiguity in the categorical variables. Nevertheless, we believe that our findings may enable clinical oncologists to better select LCT for patients with oligometastatic NSCLC. We have launched a phase II study to further explore the role of LCT for oligometastatic NSCLC after first-line systemic treatment; this study commenced in 2018 (Chinese Thoracic Oncology Group, CTONG 1602).

## Conclusions

In conclusion, we used the results of our single-center study to create a stratification model that would predict the effects of LCT on oligometastatic NSCLC. The results indicate that smokers with T3/4 oligometastatic NSCLC may not benefit from LCT. However, future studies are needed to explore the genetic signatures of patients who may benefit from LCT for oligometastatic disease.

## Data Availability

The datasets used and/or analyzed during the current study are available from the corresponding author on reasonable request.

## Abbreviations

LCT: Local consolidative treatment
NSCLC: Non-small cell lung cancer
OS: Overall survival
PFS: Progression-free survival
